# Microstructure and Wear Property of ZrO_2_-Added NiCrAlY Prepared by Ultrasonic-Assisted Direct Laser Deposition

**DOI:** 10.3390/ma14195785

**Published:** 2021-10-03

**Authors:** Zhengyao Yi, Chenchen Song, Guohui Zhang, Tianqi Tong, Guangyi Ma, Dongjiang Wu

**Affiliations:** 1 School of Navigation and Naval Engineering, Dalian Ocean University, Dalian 116023, China; yizhengyao@163.com; 2 Key Laboratory of Environment Controlled Aquaculture of Ministry of Education, Dalian Ocean University, Dalian 116023, China; 3 Key Laboratory for Precision and Non-Traditional Machining Technology of Ministry of Education, Dalian University of Technology, Dalian 116024, China; song.chenchen@mail.dlut.edu.cn (C.S.); guohui-zhang@foxmail.com (G.Z.); djwudut@dlut.edu.cn (D.W.); 4 School of Engineering and Technology, University of Sydney, Sydney 2006, Australia; tton6391@uni.sydney.edu.au

**Keywords:** direct laser deposition, ultrasonic-assisted, ZrO_2_-added NiCrAlY, microstructure, wear properties

## Abstract

For improving the wear properties of NiCrAlY, the 10 wt %, 20 wt % and 30 wt % ZrO_2_-added NiCrAlY samples were prepared by ultrasonic-assisted direct laser deposition, respectively. The results showed that the ultrasonic-assisted direct laser deposition can realize the ZrO_2_-added NiCrAlY preparation. Furthermore, due to the cavitation effect and agitation of the ultrasound in the molten pool, ultrasonic-assisted could make the upper surface of the samples smoother and flatter, and it also improved the microstructural homogeneity. The microstructure was mainly composed of columnar dendrites, and most of ZrO_2_ particles were located in the intergranular regions. The principal phase constituents were found to contain γ-Ni and t-NiZr_2_, and the amorphous (Ni, Zr) intermetallic phase generated, because of more rapid solidification after ultrasound assisted. The microhardness was improved slightly with the increase of ZrO_2_ contents, rising from 407.9 HV (10% ZrO_2_) to 420.4 HV (30% ZrO_2_). Correspondingly, wear mass loss was decreased with the maximum drop 22.7% of 30% ZrO_2_ compared to that of 10% ZrO_2_, and wear mechanisms were mainly abrasive wear with slightly adhesive wear. After applying ultrasound, the oxide islands in samples disappeared, and more ceramic particles were retained. Thus, the hardness and wear performance of the samples were improved.

## 1. Introduction

MCrAlY (M means Ni and/or Co) is an engineering coating material with excellent properties. It has the advantages of good wear resistance, excellent high temperature oxidation resistance, and strong corrosion resistance [1,2,3]. In general, MCrAlY is extensively used in turbos, blades, and other hot-end parts of aero engines and large gas turbines [4,5]. However, with the rapid development of aerospace and energy, the working temperature of hot-end components is getting higher. And the MCrAlY coatings have been difficult to meet the working conditions of high-temperature environment. Thus, the coatings with CeO_2_, Al_2_O_3_, or ZrO_2_ adding to MCrAlY matrix are developed to increase its high-temperature corrosion resistance and thermal stability [6,7,8]. At the same time, there are also two-layer coating with MCrAlY as a bonding layer with a ceramic layer on the surface [9], and graded thermal barrier coating with bonding metal and ceramic composites in the middle [10,11]. Furthermore, this material has important guiding significance for the preparation of graded thermal barrier coatings.

At present, the main preparing methods of ceramic-added MCrAlY and graded thermal barrier coatings are thermal spraying (including plasma spraying, flame spraying and arc spraying), transfer arc cladding, and laser cladding. The thermal spraying method can simply operate, and shows high working efficiency. However, the sprayed materials are mechanically bonded to its substrate, and a large number of pores exist in the microstructure [12]. Hence, the density cannot be guaranteed. Transferred arc cladding and laser cladding can realize metallurgical bonding between coatings and substrate, and the density can be effectively improved. Unfortunately, owing to the poor wettability of MCrAlY and ceramics, it is easy to form ceramic agglomerates and make them non-uniformly distributed.

Bolelli G. prepared NiCrAlY + Al_2_O_3_ coatings by plasma spraying coupled with feeding of dry powder plus suspension, and measured the friction properties at different temperatures of the coating materials [13]. However, it is still laminated structural, and a mass of inter-layer voids may affect its wear resistance. Demian C. used a plasma transfer arc to prepare NiCrAlY + ZrO_2_ coatings, then examined and analysed the microstructure [14]. It was found that the microstructure had large ceramic oxide islands in the matrix, resulting in a heterogeneous microstructure. Fortunately, Li prepared WC-Ni composite coatings by laser cladding on Ti-6Al-4V alloy substrates under high frequency micro-vibration [15]. The vibration made the coating microstructure more uniform, and its microhardness and wear performance were significantly improved. Liu fabricated Ni60CuMoW coatings by combination of laser cladding and mechanical vibration processing [16]. With the help of mechanical vibration, he found that the corrosion and wear properties of the coating were significantly increased. Li successfully fabricated TiC/AlSi10Mg alloys by laser additive manufacturing under high-frequency micro-vibration [17]. The high-frequency micro-vibration could accelerate the melt flow, promote the floating of gas and slag in the molten pool and significantly reduce the pores in the alloys, which could obtain refined and homogenous microstructure with superior alloy density as well as good performances.

In summary, ultrasonic-assisted direct laser deposition technology has many advantages in the preparation of metal-ceramic composites. Direct laser deposition is a kind of additive manufacturing technology. Compared with traditional subtractive processing methods, this method has the unique advantages of additive manufacturing technology such as high material utilization rate, easy preparation of complex parts, and short processing cycle [18,19,20], and it is suitable for the preparation of gradient materials. Ultrasound is applied at the bottom of the substrate; the ultrasound can effectively convey the vibration to the molten pool, utilizing the cavitation effect, acoustic flow effect, and stirring effect of the ultrasonic wave to improve the microstructure and properties of the formed material. Using this method to prepare ZrO_2_-added NiCrAlY material can obtain a more uniform ceramic distribution as well.

The ZrO_2_-added NiCrAlY coating was prepared by ultrasonic-assisted direct laser deposition. Macroscopic morphology, microstructure, and phase of the samples prepared with ultrasonic-assisted were evaluated. The effects of different ZrO_2_ content on microstructure and mechanical properties were analyzed.

## 2. Experimental Procedures

**Figure 1:**
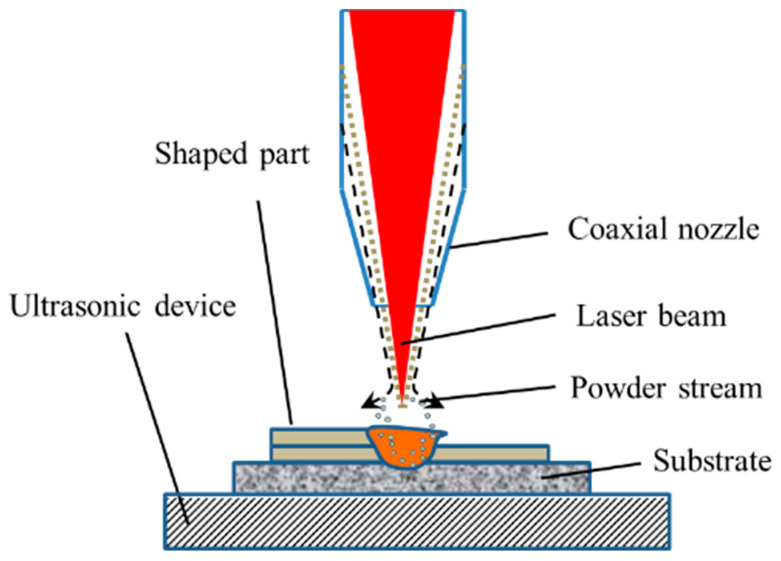
Working principle of the equipment.

The working principle of the direct laser deposition system used in this experiment is shown in Figure 1. The system includes Nd:YAG continuous laser device (JK1002, GSI Lumonics, Rugby, England.) and a three-hopper powder feeder(DPSF-D3, Beijing Aeronautical Manufacturing Technology Research Institute, Beijing, China) which can realize various powder mixing and conveying. The ultrasonic vibration system is a standard sine wave generator with frequency of 20 kHz (the material of ultrasonic vibration plate is stainless steel). The ultrasonic vibration plate is rectangular, and the ultrasonic vibration direction is vertical. The substrate is pressed on the ultrasonic vibration plate by the fixture, and the guided wave medium is filled between the substrate and the ultrasonic vibration plate to ensure the effective transmission of ultrasonic. The experiment used 99.9% high-purity argon as the shielding gas and carrying gas.

The experiment used an Inconel718 substrate with a thickness of 12 mm. Before the experiment, sandpaper was used to polish the substrate surface to remove impurities and polish the flat surface, and then the surface was cleaned by ethanol. The particle sizes of Ni-22Cr-10Al-1Y and YSZ (Y_2_O_3_ stabilized ZrO_2_) powders used in the research were both 45–90 μm (Beijing Sunspray New Materials Co., Ltd., Beijing, China). The chemical composition of the powders was shown in Table 1 and Table 2. Three combinations (by mass fraction) of NiCrAlY-ZrO_2_ composites were designed, as 90%NiCrAlY + 10%ZrO_2_, 80%NiCrAlY + 20%ZrO_2_, 70%NiCrAlY + 30%ZrO_2_ (abbreviated to 10%ZrO_2_, 20%ZrO_2_, and 30%ZrO_2_ in later sections). The formed sample size was 16 mm square with a thickness of 1.8 mm. Although ZrO_2_ powder had higher melting point, its absorption rate for laser was higher than that of metal, so the forming process parameters of the two materials were not much different. After many process explorations, the main process parameters were shown in Table 3, it could be used to fully melt two kinds of powders. Two sets of experimental samples were formed with the same process parameters. One set was not ultrasound-assisted, and the other one was assisted by ultrasonic vibration with the power of 90 W. During the depositing process, the scanning directions were crossed between adjacent layers to relieve the thermal stress.

After deposited, the samples were cut along their longitudinal sections by wire cutting, and then they were ground, polished, and etched by Kelling’s No.2 reagent. The microstructure of the sample was observed by optical microscope (MX40F, Olympus, Tokyo, Japan) and scanning electron microscope (Supra55, Zeiss, Jena, Germany). The elemental analysis was performed by EDS testing. The phase compositions of these samples were analyzed by X-ray diffraction (Empyrean X, PANalytical, Almelo, Netherlands) with Cu-Ka radiation at a scanning angle of 30° to 90°. Vickers (MVS-1000Z, Xutai Instrument Technology, Hefei, China) was employed for microhardness tests with a 200 g load for 15 s. In addition, eight measure points were applied on each sample, and the averages and standard deviations were calculated. The wear properties of each sample were tested at room temperature using a universal friction and wear tester (MMW-1A, Jinan PuYe Mechanic & Electronics Technology, Jinan, China). The testing parameters were as follows: load 20 N, friction time 15 min, turntable speed 100 r/min. After the friction test, wear morphology was observed by a confocal microscope (VK-X100/X200, Keyence, Osaka, Japan).

## 3. Results and Discussion

### 3.1. Macroscopic Morphology

**Figure 2:**
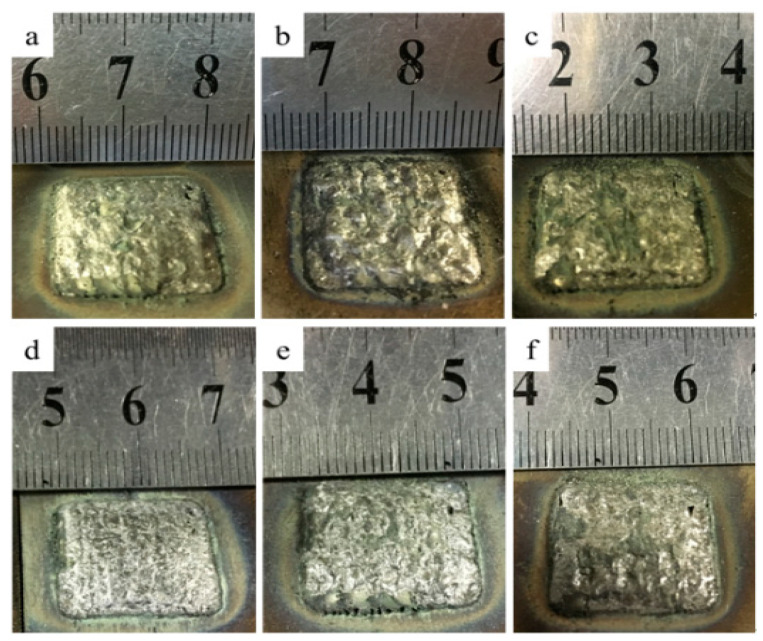
Macro-morphology of NiCrAlY-ZrO_2_ composites samples with oxide scale swept off. (**a**) 10% ZrO_2_ composites without ultrasonic vibration, (**b**) 20% ZrO_2_ composites without ultrasonic vibration, (**c**) 30% ZrO_2_ composites without ultrasonic vibration, (**d**) 10% ZrO_2_ composites with ultrasonic vibration, (**e**) 20% ZrO_2_ composites with ultrasonic vibration, (**f**) 30% ZrO_2_ composites with ultrasonic vibration.

With no ultrasonic assistance, the prepared samples have oxide scales falling off from the top during the cooling process. With the increase of ZrO_2_ content, the amount of oxide scales that broke from the sample increased slightly. After swiping off the scales with a scraper, the upper surface of the sample was found to be inferior in forming quality, poor in flatness, showing local concave and local convex (Figure 2a–c).

The melting point of ZrO_2_ is higher than that of the metal matrix. Therefore, ZrO_2_ first solidifies during the rapid cooling, but the molten liquid nickel-based alloy and ZrO_2_ have poor wettability [21]. This causes a large amount of oxide scales peeling, and this phenomenon becomes more remarkable as the increase of ZrO_2_ addition. More seriously, during the solidification, it appears the phenomenon of macroscopic separation of ceramic and metal matrix. Furthermore, under the huge thermal physical property differences between ceramic and metal, a large residual stress generates during the process and force the ceramic to burst off from the upper. Therefore, it can be judged that the main content of the oxide scale is ZrO_2_.

When the ultrasound was applied, the oxide scales still peeled off from the surface. However, after the oxide scales were cleaned up, the top surface quality of the sample was significantly improved, and the local concave and convex were reduced (Figure 2d–f). The main reason for the flatness improvement is that the ultrasonic has stirring effect on the molten pool, then the large oxide scale has less chance to accumulate on the local upper surface, thus the concave and convex caused by large oxide scales peeling off are reduced. In addition, the ultrasonic can lower the surface tension gradient, and the spheroidization phenomenon can be weakened, so that the adjacent scanning tracks are more even during the depositing process.

### 3.2. Oxide Islands

**Figure 3:**
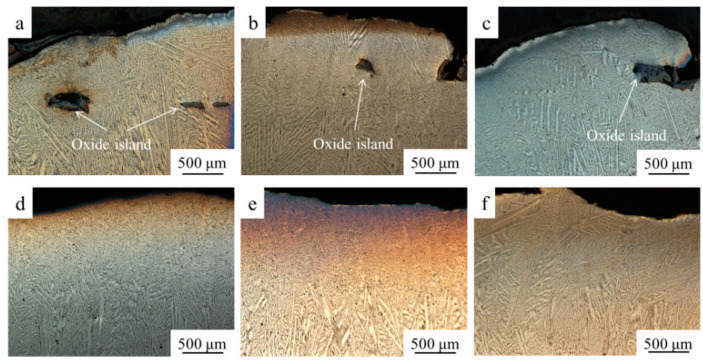
Microstructure of NiCrAlY-ZrO_2_ composites sample in low magnification. (**a**) 10% ZrO_2_ composites without ultrasonic vibration, (**b**) 20% ZrO_2_ composites without ultrasonic vibration, (**c**) 30% ZrO_2_ composites without ultrasonic vibration, (**d**) 10% ZrO_2_ composites with ultrasonic vibration, (**e**) 20% ZrO_2_ composites with ultrasonic vibration, (**f**) 30% ZrO_2_ composites with ultrasonic vibration.

Figure 3a–c shows the cross-section images of the samples prepared without ultrasonic assisted. It can be seen that there are black blocks embedded in the metal matrix; they are large in size and irregular in shape, and judged as oxide islands.

As the melting point of ZrO_2_ is higher than that of NiCrAlY (about 1450 °C), hence, the ceramic first precipitates from the molten pool during the solidification process. When the ZrO_2_ particles are just precipitated with a small size, these particles circulate in the molten pool. Therefore, the first precipitated ceramic particles move in the liquid metal and collide with other particles at a high frequency. When the ZrO_2_ particles meet together in the liquid, they mechanically bond together by laser remelting to form a metallurgical bonding, ultimately forming an irregularly shaped oxide islands. Once they get larger in size, there are more chances to collide with small particles, and there are more forces to attract small particles to agglomerate.

However, the density of ZrO_2_ is only 5.6 g/cm^3^, quite lower than the density of NiCrAlY alloy (approximately 8.3 g/cm^3^). As a result, the formed oxide islands tend to float in the liquid metal. Most of the aggregated ceramic also float to the surface. After they are completely cooled, due to the huge stress caused by the large difference of thermal expansion coefficient of the metal and the ceramic, the ceramic starts to peel off, forming the oxide scales. Further, a small number of oxide islands fail to float to the surface in a short time, thus, solidified in the metal matrix.

Figure 3d–f shows the microstructure after ultrasonic vibration applied. There are no clear massive oxide islands in the matrix, so the ultrasonic vibration has a clear effect on improving the microstructure uniformity. There are two reasons for the removal of oxide islands:

Firstly, the ultrasonic vibration has a cavitation effect on the metal melt. Ultrasonic vibration will form a hollow bubble in the negative pressure zone of the melt, which will produce tiny explosions under the high frequency vibration and pressure changes [22]. This will break up the mechanically bonded large oxide islands and make them become small ceramic particles distributed in the microstructure [23,24,25]; Secondly, the ultrasonic vibration has an acoustic flow effect and mechanical stirring effect on the melt. Thus, the ceramic particles will increase the chance of colliding with other particles, and then to form larger particles [25]. According to the Stokes’ law, under the ultrasonic vibration, the fluidity of the metal liquid increases and the viscosity decreases, increasing the floating speed. Meanwhile, some cavitation bubbles generated by the cavitation effect adhere to the oxide islands, thereby reducing the density of the oxidation islands and increasing the floating speed [26]. Therefore, the oxide islands float upward in a shorter time to ensure high uniformity of the microstructure.

### 3.3. Microstructure

**Figure 4:**
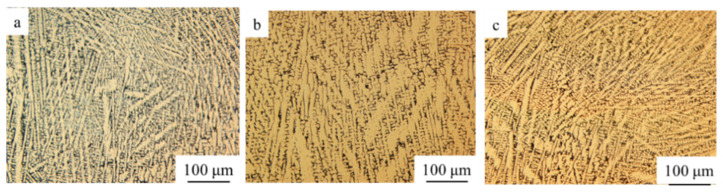
Microstructure of different compositional NiCrAlY-ZrO_2_ samples: (**a**) 10% ZrO_2_, (**b**) 20% ZrO_2_, (**c**) 30% ZrO_2_.

Figure 4 shows the microstructure of different compositions prepared with ultrasonic-assisted. It can be seen that the microstructure of each sample is similar. They all have columnar dendrites morphology, and the growth directions are generally along the deposition direction of the sample. However, in the local area, some columnar dendrites are of disordered growth directions. This phenomenon of disordered dendrites has been found in the study of laser cladding of NiCrAlY coatings [27,28].

**Figure 5:**
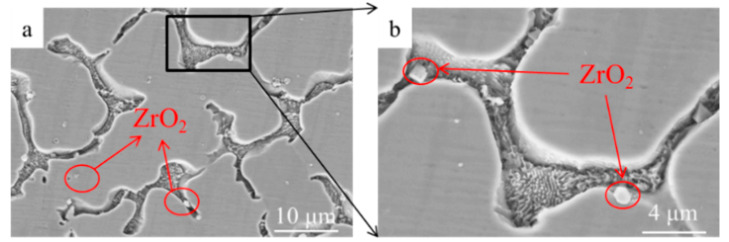
SEM images of the microstructure of 20% ZrO_2_: (**a**) distribution image of ZrO_2_, (**b**) enlarged image of a local area of (**a**).

When using high magnification scanning electron microscope, white particles can be observed uniformly distributed in the all three composition samples. According to the EDS results, these particles are ZrO_2_ phase. The ceramic particles are mostly distributed in the grain boundaries and a few in the grain matrix, as shown in Figure 5. Owing to the poor wettability of ceramic and metal, the particles cannot easily adhere to the dendrites during the solidification process, and they are pushed by the growing dendrites to the grain boundaries. Therefore, the ceramic particles concentrated at the grain boundaries are more than the particles wrapped in the grain matrix.

**Figure 6:**
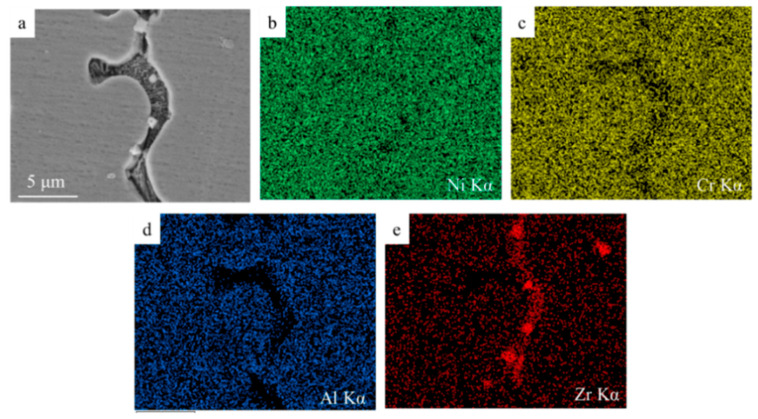
Elements distribution map of 20% ZrO_2_ sample: (**a**) microstructure of 20% ZrO_2_ sample, (**b**–**e**) element surface scan results for region (**a**).

In Figure 5b, it can be clearly seen that discontinuous reticulate precipitates in the grain boundaries. As shown in Figure 6, the intergranular precipitates are rich in Zr element, whereas the Al and Cr elements are mostly concentrated in the intragranular area. When the size of the solute atom is similar to that of Ni [29], it has a greater solubility in the Ni matrix, so Al and Cr can be dissolved in the Ni matrix, whereas the Zr element has only 3% solubility. This result also verifies the distribution of ZrO_2_ particles and the enrichment of Zr element in intergranular area. Thus, it is judged that intergranular precipitates are a kind of (Ni, Zr) intermetallic compound.

### 3.4. Phase Patterns

**Figure 7:**
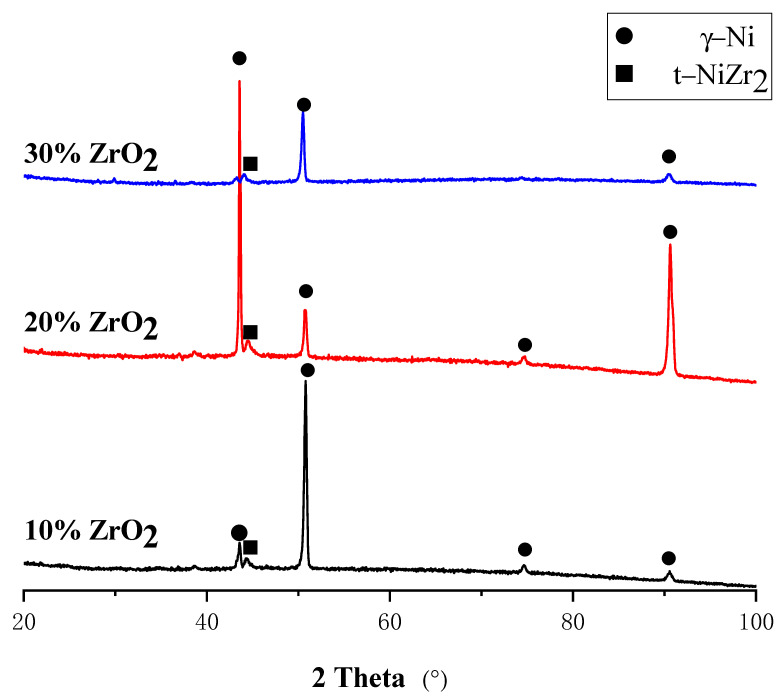
XRD patterns of different compositions of NiCrAlY-ZrO_2_ composites.

**Figure 8:**
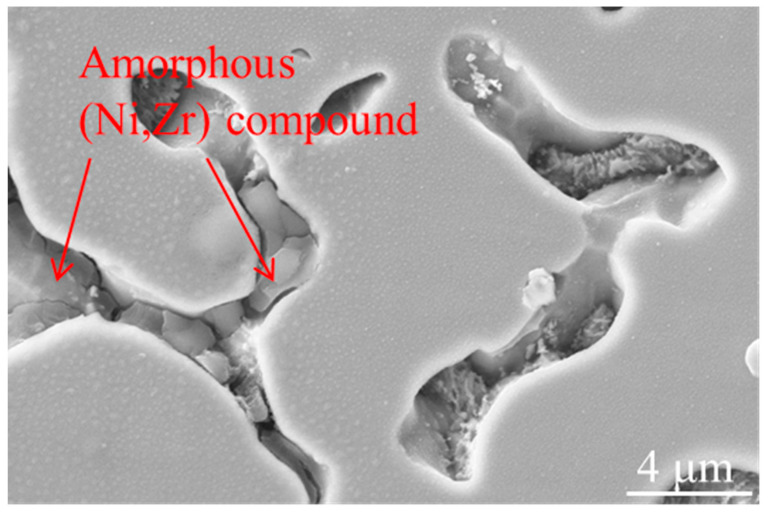
Amorphous (Ni,Zr) intermetallic compound at grain boundary.

The XRD results of the ZrO_2_-added NiCrAlY with different composition ratios are shown in Figure 7. It can be seen that the three coatings contain the same phases, mainly γ-Ni phase and t-NiZr_2_ phase. However, it did not find the (Ni, Zr) intermetallic compound precipitated in the intergranular—this is because of the tiny quantity of intergranular precipitates.

(Ni, Zr) intermetallic compounds have glass forming ability. Under the conditions of rapid solidification, they can form amorphous substances with disordered arrangement of atoms [30]. In Figure 8, a tiny amount of area containing glassy (Ni, Zr) compounds was found. The forming reason of the amorphous compounds may ascribe to the instantaneous cavitation high temperature and high temperature gradient induced by ultrasonic-assisted [31,32]. Since the content of the formed glassy material is quite low, the overall performance of the prepared NiCrAlY-ZrO_2_ composites will not be affected. This coexistence of amorphous and crystalline intermetallic compounds has also been reported in other Ni-Zr/Zr-Si laser cladding research [33].

### 3.5. Mechanical Properties

**Figure 9:**
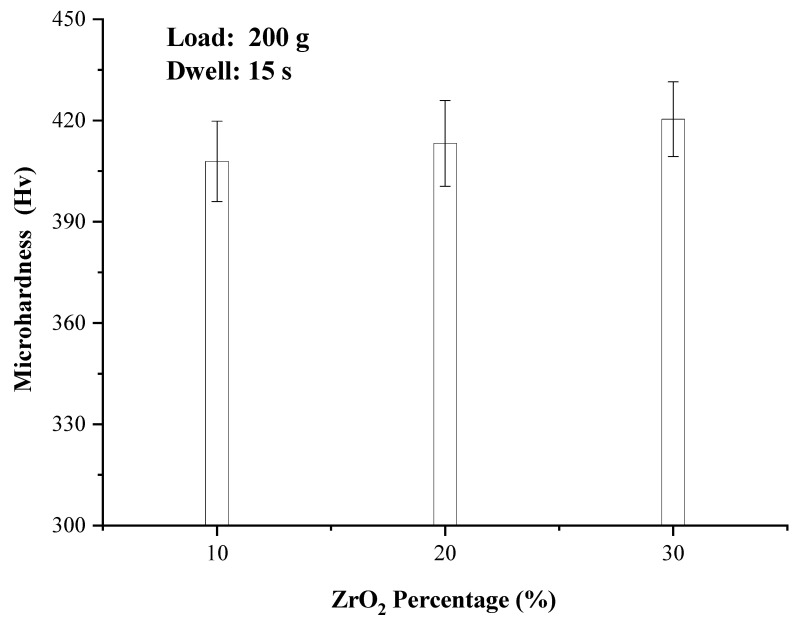
Microhardness of different ZrO_2_ compositions.

In the microhardness test, 8 points were taken for each sample, and the mean value and standard deviation were calculated respectively. As shown in Figure 9, the microhardness of the composites increases slightly with increasing ZrO_2_ content, from 407.9 HV of 10% ZrO_2_ to 413.2 HV of 20% ZrO_2_, and finally to 420.4 HV of 30% ZrO_2_. ZrO_2_ is a ceramic phase and its microhardness is approximately 1500 HV. According to the lever principle of composite materials, the microhardness of NiCrAlY-ZrO_2_ composites should be greatly improved. However, in this study, the microhardness is slight growth. The main reason is that part of the added ZrO_2_ is lost in the form of oxide scale due to poor wettability and large density difference, and the other part of ZrO_2_ reacts to form the NiZr_2_ intermetallic compound. Only part of ZrO_2_ remains in the metal matrix to play a role of hardness strengthening, so the microhardness of the composite has little change.

As summarized in Table 4, the wear loss weight decreases with the increasing ZrO_2_ content, thus the wear resistance increases. According to the analysis of Archard model:

*V* = *KPL*/*H* (1)

where *V* is the wear loss volume, *K* is the wear coefficient, *P* is the pressure between the sample and the friction, *L* is the relative slip distance, and *H* is the hardness of the sample. In the present friction–wear test, both *P* and *L* are set to be the same. From the above, it can be seen that the hardness of the three composites increases slightly with more ZrO_2_ added. Therefore, the wear mass loss is significantly reduced. It can be concluded that the wear resistance of ZrO_2_-added NiCrAlY composites increase with more ZrO_2_ content.

**Figure 10:**
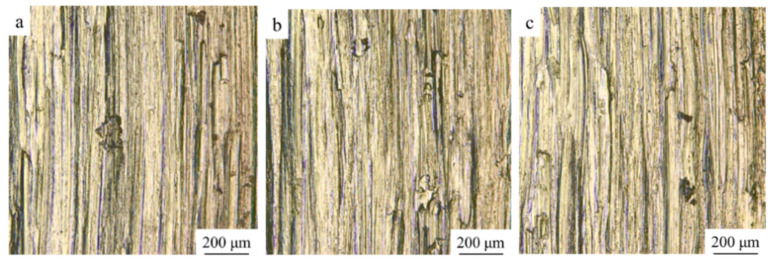
Wear morphology: (**a**) 10% ZrO_2_, (**b**) 20% ZrO_2_, (**c**) 30% ZrO_2_.

As illustrated in Figure 10, there is no significant difference in the wear surface morphology of the three samples. The main wear mechanism is abrasive wear with slight adhesive wear. There are many furrows caused by abrasive wear on the wear surface. Since the hardness of the 45-steel friction pair after quenching is greater than the sample, the 45-steel wear debris will repeatedly be squeezing and scratching the surface of the sample during the wear process, thereby creating the furrows. The sample is doped with ceramic, so that friction parts are poor in intersolubility with each other, and are less prone to adhesive wear. A small amount of the adhesive points on the wear surface can also prove this. Meanwhile, ceramic particles are randomly distributed in the microstructure, which may cause the discontinuity of the γ-Ni matrix. Thus, it is beneficial to improve the plastic deformation-resistance of the metal matrix. In addition, the sparsely distributed ZrO_2_ ceramic particles also withstand friction, directly reducing the wear area of the metal matrix. Furthermore, when there are more ceramic particles dispersed in the matrix, the higher the wear resistance is.

After employing the ultrasonic-assisted process, the defects such as oxidation islands in the microstructure disappear, increasing the density of the sample. In addition, the agitation of the molten pool by ultrasound facilitates rapid solidification. Therefore, this promotes grain refinement and uniform distribution of ceramic particles. It can be considered that the application of ultrasonic energy can effectively improve the wear resistance of the sample.

## 4. Conclusions

This study investigated the macroscopic morphology, microstructure, microhardness, and friction-wear of ZrO_2_-added NiCrAlY by ultrasonic-assisted direct laser deposition process. The conclusions are as follows:

- Ultrasonic-assisted could make the upper surface smoother and flatter. Ultrasonic sound flow, cavitation and other effects on the molten pool could effectively reduce or remove the ceramic oxide islands in the matrix, further to improve the microstructural homogeneity of the ZrO_2_-added NiCrAlY;
- The microstructure of 10% ZrO_2_, 20% ZrO_2_, and 30% ZrO_2_ added NiCrAlY were similar—mainly composed of columnar dendrites. The growth directions were generally along the deposition direction, and there were partially disordered dendrites. ZrO_2_ ceramic particles were randomly distributed in the microstructure, most of which were located in the intergranular regions, with a small number wrapped in the grain matrix;
- The three ZrO_2_ -added NiCrAlY samples had the similar phase patterns, mainly contained γ-Ni phase and t-NiZr_2_ phase. (Ni, Zr) intermetallic phase was also generated in the process. The fast-cooling speed caused by the ultrasonic-assisted process could induce the formation of (Ni, Zr) intermetallic compound;
- The microhardness of the composites improved slightly with the increase of ZrO_2_ content, rising from 407.9 to 420.4 HV. The main reason is that part of the added ZrO_2_ is lost in the form of oxide scale, and part of ZrO_2_ forms NiZr_2_ intermetallic compound, and a small amount of ZrO_2_ remains in the metal matrix to strengthen the hardness, so the microhardness of the composite has little change;
- The wear resistance of the three composites increased with the increase of ZrO_2_ content. The values of wear mass loss declined, as the maximum drop 22.7%. The wear mechanism for the three composites was mainly abrasive wear with slight adhesive wear. There is no obvious change in the wear mechanism of different composites.

After the preparation of ZrO_2_-added NiCrAlY composite was preliminarily achieved by the ultrasonic-assisted process, the influences of different ultrasonic parameters on ZrO_2_-added NiCrAlY composite will be studied in the next step to further optimize the microstructure of the composite. To improve the columnar dendritic morphology, the wettability of NiCrAlY and ZrO_2_, and the mechanical properties of the material.

## Figures and Tables

**Table 1:** Composition and contents of NiCrAlY powders.

NiCrAlY	Ni	Cr	Al	Y
Content (wt.%)	67.70	21.65	9.94	1.08

**Table 2:** Composition and contents of ZrO_2_ powders.

YSZ	ZrO_2_	Y_2_O_3_	HfO_2_	TiO_2_	I
Content (wt.%)	86.42	7.13	6.24	0.13	0.05

**Table 3:** Process parameters of NiCrAlY-ZrO_2_ composites.

Laser Power *P* (W)	Scanning Speed *V* (mm/min)	Powder Feeding *Q* (g/min)	Layer Thickness Δ*Z* (mm)
560	300	2.3	0.3

**Table 4:** Wear mass loss of NiCrAlY-ZrO_2_ composites.

Content	Wear Mass Loss (g)	Changing Percentage (%)
0% ZrO_2_	0.022	0
20% ZrO_2_	0.020	−9.1
30% ZrO_2_	0.017	−22.7

## Data Availability

Data sharing not applicable.
